# Extremely high high‐density lipoprotein cholesterol with coronary artery disease: Case report

**DOI:** 10.1002/ccr3.4092

**Published:** 2021-05-04

**Authors:** Khalid Sawalha, Gilbert‐Roy Kamoga

**Affiliations:** ^1^ Internal Medicine Division White River Health System Batesville AR USA

**Keywords:** alcohol abuse, cholesteryl ester transfer protein, coronary artery diseases, high‐density lipoprotein, lipoproteins

## Abstract

Whether this extremely high high‐density lipoprotein (HDL) level due to chronic alcohol abuse or cholesteryl ester transfer protein or others, we report this interesting case of extremely high high‐density lipoprotein to emphasize that serum HDL is not always protective from development of coronary heart disease.

## Graphical abstract

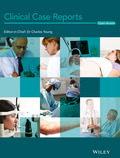

## 1 INTRODUCTION

A 52‐year‐old white female with past medical history of hypertension, heart failure preserved ejection fraction, anxiety, and alcohol abuse presented to the emergency department with chest pain. Her cardiac markers along with serial electrocardiograms and myocardial perfusion scan did not reveal any reversible coronary ischemia. Left heart catheterization showed nonobstructive coronary artery disease with 50% stenosis of the right coronary artery. Upon further testing, she was found to have extremely high high‐density lipoprotein level of 218 mg/dL (39‐61). Whether this extremely high high‐density lipoprotein (HDL) level due to chronic alcohol abuse or cholesteryl ester transfer protein (CETP) or others, we report this interesting case of extremely high high‐density lipoprotein to emphasize that serum HDL is not always protective from development of coronary heart disease.

High‐density lipoprotein cholesterol is a biomarker inversely associated with an increased risk of coronary heart disease (CHD) events. It is of considerable value in assessing patients’ coronary artery disease risk. However, low levels of HDL cholesterol independent of non‐HDL cholesterol have not been shown to increase risk of CHD. A 2009 meta‐analysis of 108 randomized trials involving nearly 300 000 patients at risk of cardiovascular events found no association between treatment‐induced increases in HDL cholesterol with risk ratios for CHD deaths, CHD events, or total deaths after adjustment for changes in low‐density lipoprotein (LDL) cholesterol.^1^

High‐density lipoprotein is a complex circulating particle with many subspecies that vary in lipid and protein composition.^2^ Cholesterol is a major component of the particle, and the amount of cholesterol contained in HDL particles can be directly measured; it is referred to as HDL cholesterol. In clinical practice, non‐HDL cholesterol, rather than HDL cholesterol, is used to risk stratify patients. On the other hand, extremely high levels of HDL have been associated with high cause mortality in men and women.^3^ Therefore, we share this interesting case of extremely high HDL level with its differentials.

## 2 CASE PRESENTATION

A 52‐year‐old white female with past medical history of hypertension, heart failure with preserved ejection fraction, generalized anxiety disorder, and alcohol dependence/abuse presented to the emergency department with retrosternal chest pain associated with nausea. She has family history significant for hypertension, diabetes mellitus type II, and coronary artery disease. Her home medications include the following: venlafaxine 75 mg, metoprolol tartrate 12.5 mg, and zolpidem 10 mg. Patient reports long history of 10 years or more of alcohol abuse reported as half a bottle of vodka or whiskey on daily basis. Vitals upon admission were blood pressure 155/100 mm Hg and a heart rate of 110 beats per minute. Her electrocardiogram showed sinus tachycardia with no acute ST‐T wave changes. Initial troponin level was negative <0.012 (ng/mL). She was admitted for observation in the progressive care unit. Further laboratory data showed triglyceride 122 mg/dL (0‐149), total cholesterol 265 mg/dL (107‐200), LDL 41 mg/dL (0‐99), HDL 218 mg/dL (39‐61) with repeated level of 200 mg/dL. Her hemoglobin A1C was 5.3% (0‐5.99), TSH 1.67 uIU/mL (0.465‐4.68), folic acid >20.0 ng/mL (2.76‐19), and vitamin B12 of 197.0 pg/mL (239‐931). Other laboratory data including complete blood count and comprehensive metabolic panel were within normal limits. The trend of cardiac markers was negative. She had myocardial perfusion scan done showing no reversible ischemia. However, due to persistent chest pain, the decision was made to send the patient for left heart catheterization which showed nonobstructive coronary artery disease with 50% stenosis in the right coronary artery. She was discharged home on omega‐3 fatty acids eicosapentaenoic acid (EPA) and docosahexaenoic acid (DHA) 1000 mg folic acid 1 mg, atorvastatin 40 mg, aspirin 81 mg, and thiamine 100 mg with recommendations for further lipidology and genetic testing.

## 3 DISCUSSION

High serum HDL cholesterol (>60 mg/dL [1.6 mmol/L]) may be hereditary or associated with conditions such as alcohol abuse, hypothyroidism, phenytoin treatment, and insulin treatment in type 1 diabetes or as a result of regular moderate to intense aerobic exercise. It is usually associated with a lower risk of coronary heart disease (CHD).^4^ Several studies have shown that high HDL cholesterol levels are associated with an increased risk of atherosclerosis and cardiovascular events.^5^ In these situations, HDL particles are dysfunctional in their antiatherogenic properties.^6^ Large HDL particles have a reduced content of anti‐inflammatory proteins and lipids that may account for their dysfunctional properties. However, the question is whether the HDL particles are functional in patients with high HDL cholesterol levels. In one series of patients with elevated HDL cholesterol levels who had CHD, it was found that the HDL particles were functionally impaired with regard to antioxidant and anti‐inflammatory activities.^5^

Our patient had an extremely high serum HDL lipoprotein levels documented on two occasions, and despite the high HDL levels, she had evidence of coronary artery disease although nonobstructive. The first on our differential was her history of chronic alcohol abuse/dependence. Her long history (10 years or more) of alcohol abuse reported as half a bottle of vodka or whiskey on daily basis is likely responsible for her elevated levels of HDL as alcohol consumption was reported to increase HDL cholesterol levels.^7^ From the literature review, it is possible that she has dysfunctional HDL particles that do not confer the protection against CHD that functional HDL particles would. Thus, assessment of HDL function among those with high HDL levels may be an important step in risk assessment for CHD to provide further clinical guidance for our patients.

Second on our list is CETP deficiency as it is integrally involved in HDL metabolism through mediating the transfer of cholesteryl esters from HDL particles to the triglyceride‐rich lipoproteins LDL and very low‐density lipoprotein (VLDL). Polymorphisms affecting the activity of CETP, such as an isoleucine for valine substitution at codon 405 (I405V), are common. In a study from Denmark, for example, 43 percent of people studied were heterozygous for I405V and 11 percent were homozygous for I405V.^8^ Polymorphisms such as I405V that reduce the activity of CETP typically increase plasma HDL cholesterol concentrations.^9^, ^10^, ^11^ Although we did not test the patient as inpatient, but upon discharge recommendations were for further lipidology and genetic testing as an outpatient.

Other differentials such as hypothyroidism, phenytoin treatment, and insulin treatment in type 1 diabetes or as a result of regular moderate to intense aerobic exercise were ruled as the patient has TSH level, not on phenytoin or insulin treatment and not active.

## 4 CONCLUSION

We share this interesting case of extremely high serum HDL and how it is not always protective from development of CHD. It has been demonstrated that extremely high levels of serum HDL in both men and women in the general population have high all‐cause mortality. Increased risk of CHD is thought to be from dysfunctional HDL particles that maybe more prevalent in individuals with high serum HDL levels. Thus, we should not assume that a high HDL in our patients is always protective.

## CONFLICT OF INTEREST

None declared.

## AUTHOR CONTRIBUTIONS

KS: Writing the manuscript and reviewing it; GRK: Reviewing the manuscript.

## ETHICAL APPROVAL

Our institution does not require ethical approval for reporting individual cases or case series.

## INFORMED CONSENT

Verbal consent was obtained directly from the patient.

## Data Availability

Data are available upon request.

## References

[1] Briel M , Ferreira‐Gonzalez I , You JJ , et al. Association between change in high density lipoprotein cholesterol and cardiovascular disease morbidity and mortality: systematic review and meta‐regression analysis. BMJ. 2009;338:b92. 19221140 10.1136/bmj.b92 PMC2645847

[2] Rosenson RS , Brewer HB Jr , Chapman MJ , et al. HDL measures, particle heterogeneity, proposed nomenclature, and relation to atherosclerotic cardiovascular events. Clin Chem. 2011;57(3):392‐410. 21266551 10.1373/clinchem.2010.155333

[3] Madsen CM , Varbo A , Børge G , et al. Extreme high high‐density lipoprotein cholesterol is paradoxically associated with high mortality in men and women: two prospective cohort studies. Eur Heart J. 2017;38(32):2478‐2486. 28419274 10.1093/eurheartj/ehx163

[4] Rahilly‐Tierney CR , Spiro A 3rd , Vokonas P , et al. Relation between high‐density lipoprotein cholesterol and survival to age 85 years in men (from the VA normative aging study). Am J Cardiol. 2011;107(8):1173‐1177. 21296318 10.1016/j.amjcard.2010.12.015

[5] Ansell BJ , Navab M , Hama S , et al. Inflammatory/antiinflammatory properties of high‐density lipoprotein distinguish patients from control subjects better than high‐density lipoprotein cholesterol levels and are favorably affected by simvastatin treatment. Circulation. 2003;108(22):2751‐2756. 14638544 10.1161/01.CIR.0000103624.14436.4B

[6] Rosenson RS , Brewer HB Jr , Ansell BJ , et al. Dysfunctional HDL and atherosclerotic cardiovascular disease. Nat Rev Cardiol. 2016;13(1):48‐60. 26323267 10.1038/nrcardio.2015.124 PMC6245940

[7] De Oliveira E , Silva ER , Foster D , et al. Alcohol consumption raises HDL cholesterol levels by increasing the transport rate of apolipoproteins A‐I and A‐II. Circulation. 2000;102(19):2347‐2352. 11067787 10.1161/01.cir.102.19.2347

[8] Agerholm‐Larsen B , Nordestgaard BG , Steffensen R , Jensen G , Tybjærg‐Hansen A . Elevated HDL cholesterol is a risk factor for ischemic heart disease in white women when caused by a common mutation in the cholesteryl ester transfer protein gene. Circulation. 2000;101(16):1907‐1912. 10779455 10.1161/01.cir.101.16.1907

[9] Nazu A , Brown ML , Hesler CB , et al. Increased high‐density lipoprotein levels caused by a common cholesteryl‐ester transfer protein gene mutation. N Engl J Med. 1990;323(18):1234‐1238. 2215607 10.1056/NEJM199011013231803

[10] Kuivenhoven JA , de Knijff P , Boer JM , et al. Heterogeneity at the CETP gene locus. Influence on plasma CETP concentrations and HDL cholesterol levels. Arterioscler Thromb Vasc Biol. 1997;17(3):560‐568. 9102177 10.1161/01.atv.17.3.560

[11] Barzilai N , Atzmon G , Schechter C , et al. Unique lipoprotein phenotype and genotype associated with exceptional longevity. JAMA. 2003;290(15):2030‐2040. 14559957 10.1001/jama.290.15.2030

